# Effect of Simulated Gastric Acid on Surface Characteristics and Color Stability of Hybrid CAD/CAM Materials

**DOI:** 10.3390/polym17192591

**Published:** 2025-09-25

**Authors:** Handan Yıldırım-Işık, Mediha Büyükgöze-Dindar

**Affiliations:** 1 Department of Restorative Dentistry, Faculty of Dentistry, Istanbul Beykent University, 34500 Istanbul, Turkey; handanyildirim@beykent.edu.tr; 2 Department of Restorative Dentistry, Faculty of Dentistry, Trakya University, 22030 Edirne, Turkey

**Keywords:** CAD/CAM, color, gastroesophageal reflux, surface properties

## Abstract

Hybrid computer-aided design and computer-aided manufacturing (CAD/CAM) materials have gained prominence in restorative dentistry due to their advantageous mechanical and esthetic properties; however, their long-term performance may be adversely affected by acidic oral environments, such as those associated with gastroesophageal reflux disease (GERD). This in vitro study aimed to investigate the effects of simulated gastric acid exposure on the surface roughness, gloss, color stability, and microhardness of two hybrid CAD/CAM materials: Vita Enamic and Cerasmart. Standardized rectangular specimens (2 mm thickness) were prepared and polished using a clinically relevant intraoral protocol. Baseline measurements were obtained for surface roughness, gloss, color change (ΔE), and Vickers microhardness. All specimens were then immersed in hydrochloric acid (pH 1.2) for 24 h to simulate prolonged gastric acid exposure, after which the same properties were re-evaluated. Post-immersion analysis revealed significant increases in surface roughness and reductions in gloss and microhardness for both materials (*p* < 0.05), with Vita Enamic demonstrating greater susceptibility to degradation. Color changes remained below the clinically perceptible threshold, with no significant differences between materials. These findings highlight the potential vulnerability of hybrid CAD/CAM materials to acidic environments and underscore the importance of careful material selection in patients predisposed to acid-related challenges.

## 1. Introduction

Computer-aided design and computer-aided manufacturing (CAD/CAM) technology have revolutionized modern restorative dentistry by enabling the fabrication of highly esthetic and durable restorations with improved precision and efficiency. The materials used in CAD/CAM systems are broadly classified into ceramic-based and composite-based categories. While ceramic-based materials, such as feldspathic or lithium disilicate ceramics, are valued for their excellent optical properties, biocompatibility, and wear resistance, they tend to exhibit high brittleness and low tensile strength, which limit their performance under functional stress [1]. Composite-based materials, in contrast, are more RBCs and are characterized by lower elastic modulus and hardness values, thus offering ease of milling and intraoral adjustment, lower susceptibility to fracture and chipping and lower wear of opposing teeth but are often limited by their lower mechanical strength and long-term color stability [2]. To overcome these limitations, hybrid ceramics, specifically polymer-infiltrated ceramic networks (PICNs), have been developed to combine the advantageous properties of both material classes. Hybrid ceramics have garnered increasing interest due to their unique structure, combining a ceramic matrix with a polymer network. This interpenetrating phase architecture is designed to combine the favorable mechanical, esthetic, and functional attributes of ceramics with the enhanced elasticity, machinability, and reparability of resin-based materials [3,4].

Hybrid ceramics offer several notable advantages over conventional ceramics and composite resins. One of their most critical mechanical properties is their intermediate modulus of elasticity, which approximates that of natural dentin. Although reported values for the elastic modulus of dentin vary depending on measurement techniques and structural factors such as hydration and age, they typically range from 16 to 20.3 GPa [4]. In comparison, the elastic modulus of hybrid ceramics has been documented to fall within the range of 9.6 to 51.5 GPa—values that closely align with those of dentin [5,6]. This similarity facilitates more favorable stress distribution and enhances resistance to crack propagation, which is essential for the longevity of restorations [7]. Furthermore, these materials exhibit excellent milling characteristics, reduced brittleness, and do not require post-milling sintering, thereby streamlining the fabrication workflow [8]. Clinically, they demonstrate high polishability, color stability, and optical properties that are conducive to achieving superior esthetic outcomes in both anterior and posterior restorations [9].

However, despite their favorable mechanical and esthetic properties, the long-term clinical performance of hybrid ceramics largely depends on their resistance to degradation within the complex oral environment. Among the primary threats to restorative materials is dental erosion, a chemically driven process—often exacerbated by mechanical forces—that leads to the progressive loss of hard dental tissues from non-bacterial causes [10]. The etiology of dental erosion can be either extrinsic, such as from dietary acids, or intrinsic, notably due to exposure to gastric acid. Intrinsic acid exposure is commonly observed in individuals with gastroesophageal reflux disease (GERD) and in those suffering from eating disorders like bulimia nervosa [11,12]. Repeated regurgitation or vomiting exposes the oral cavity to highly acidic gastric contents (pH ≈ 1.2), posing a substantial challenge to restorative materials [13]. Such repeated chemical insult may compromise the structural and surface integrity of restorative materials, adversely affecting key properties such as surface gloss, roughness, microhardness, and color stability—parameters that are critical to ensuring the long-term functionality and esthetic outcomes of dental restorations. Given that GERD affects up to 20% of adults in industrialized countries [14], the prevalence of intrinsic acid challenges underscores the imperative to rigorously evaluate the resistance of hybrid CAD/CAM ceramics under simulated gastric acid exposure, thereby providing clinically relevant insights to guide material selection and restorative treatment planning.

Although the effects of acidic challenges on conventional ceramics and composite resins have been investigated in previous studies, there is a paucity of data regarding the behavior of hybrid CAD/CAM ceramics under simulated gastric acid exposure [15,16]. Considering the increasing adoption of these materials in contemporary restorative practice and the substantial prevalence of intrinsic acid-related oral conditions, elucidating the response of hybrid ceramics to repeated gastric acid challenges is critically important. Such knowledge is essential not only for optimizing material selection but also for informing evidence-based clinical decision-making aimed at ensuring the long-term functional and esthetic success of restorations.

Null Hypothesis (H_0_):

Simulated gastric acid exposure does not produce any statistically significant alterations in the surface gloss, surface roughness, microhardness, or color stability of hybrid ceramic CAD/CAM restorative materials.

## 2. Materials and Methods

**Figure 1:**
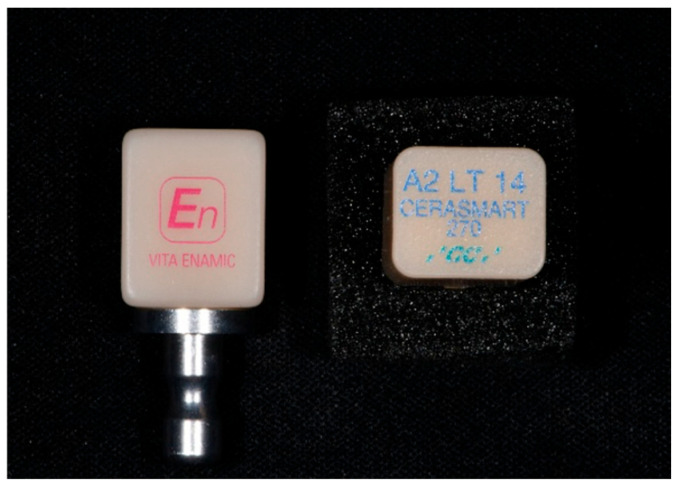
CAD/CAM blocks used in this study.

The materials used in this in vitro study, along with their respective compositions, are detailed in Table 1 (Figure 1). A priori power analysis was conducted using G*Power software (version 3.1; Heinrich Heine University, Düsseldorf, Germany) to determine the appropriate sample size. Assuming an effect size of d = 0.8, a significance level (α) of 0.05, and a statistical power of 80%, the minimum required sample size was calculated to be 25 specimens per group [17].

A total of 50 disk-shaped specimens (*n* = 25 per material group) were prepared by sectioning CAD/CAM blocks into 2 mm-thick slices using a precision cutting machine (IsoMet 1000 Precision Cutter, Buehler, Lake Bluff, IL, USA). The finishing and polishing protocol was rigorously standardized across all specimens to minimize variability. Specimens were polished sequentially following the manufacturers’ guidelines, using a system composed of Sof-Lex™ disks in descending abrasiveness (3M ESPE, St. Paul, MN, USA), Diacomp Plus Twist disks (EVE Technik, Pforzheim, Germany), and a diamond-based polishing paste (Diamond Excel, FGM, Dentscare Ltd.a, Joinville, SC, Brazil) [18]. Each polishing step was performed for 30 s under consistent pressure by a single calibrated operator. The final thickness of each specimen was verified using a digital caliper (Powerfix Electronic Digital Caliper, Padget Services, London, UK). Subsequently, all specimens were subjected to ultrasonic cleaning in distilled water for 5 min. The undersides of the samples were labeled with identification numbers and material codes using a fine-tip permanent marker. Prior to testing, all specimens were incubated in distilled water at 37 °C for 24 h.

Surface properties were evaluated both before and after exposure to simulated gastric acid. Surface roughness (Ra) was measured using a calibrated contact profilometer (Surtronic S128, Taylor Hobson Ltd., Leicester, UK) with a standard cutoff value of 0.8 mm and a stylus speed of 0.6 mm/s. Prior to each measurement, the device was calibrated using a reference block with a certified Ra value of 5.81 μm. Three readings were obtained from different regions on each specimen, and the mean value was recorded.

Surface gloss measurements were conducted using a glossmeter (Novo-Curve Rhopoint Instrumentation, East Sussex, UK) at a specular reflection angle of 60°, calibrated against the manufacturer-provided black glass standard tile (reference gloss value: 93.3 GU). Three gloss readings per sample were recorded by rotating the specimen 90° between each measurement.

Microhardness was evaluated via a Vickers microhardness tester (Tronic Digital Microhardness Tester DHV1000, Shanghai, China) using a diamond indenter under a 500 g load for 10 s [18].The diagonal length of the indentations was measured microscopically, and Vickers Hardness Numbers (VHNs) were calculated using the formula:

VMH: 1854.4 P/d2

where P is the applied load in kgf and d is the mean diagonal length in mm. The Vickers microhardness test was performed by placing three indentations on the top polished surface of each specimen, with each indentation spaced at least 1 mm apart and at least 1 mm from the specimen margins to avoid edge effects and potential stress interactions. A digital caliper and a fixed positioning protocol were used to standardize the location of indentations across all specimens. The average of the three measurements was calculated to represent the microhardness value of each specimen, thereby enhancing data reliability and reproducibility.

Color measurements were performed using a spectrophotometer (VITA Easyshade V, VITA Zahnfabrik, Bad Säckingen, Germany) under strictly standardized conditions to ensure methodological consistency. All assessments were conducted in the same location, by the same operator, using the same device, and against a standardized white background. Furthermore, to minimize the influence of diurnal variation, measurements were taken at the same time of day throughout the study. Each specimen was measured three times, and mean values were used to assess color changes.

**Figure 2:**
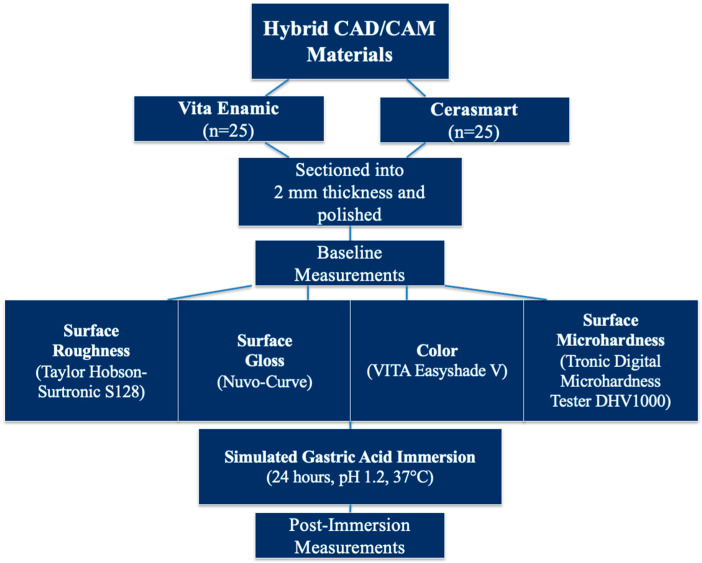
Schematic representation of the study protocol.

For the acidic challenge, specimens were immersed in simulated gastric juice at 37 °C for 24 h (Nüve incubator EN 120, Ankara, Turkey) [19]. The solution consisted of 0.113% hydrochloric acid in deionized water, adjusted to pH 1.2 (Hanna HI 83141, Hanna Instruments, Woonsocket, RI, USA) [20]. This immersion duration was equivalent to approximately eight years of gastric exposure (Figure 2). This protocol was designed to replicate the extreme acidic conditions associated with recurrent regurgitation episodes in patients with GERD or bulimia nervosa, where gastric fluid typically exhibits pH values of 1.0–1.5. The 24 h immersion period was selected based on previous reports indicating that such continuous exposure represents a worst-case clinical scenario and corresponds to approximately eight years of cumulative gastric challenge [19]. Although this in vitro model does not fully reproduce the multifactorial intraoral environment—including salivary buffering, pH fluctuations, temperature changes, and mechanical wear—it provides a standardized and reproducible method for assessing the susceptibility of hybrid CAD/CAM ceramics to intrinsic acid-induced degradation. To maintain a consistent pH, the solution was refreshed every 12 h. After exposure to the simulated gastric juice, the same tests were repeated on the other half of the specimen surface.

The color change value ΔE_Lab_ was calculated according to the following formula:

ΔE_Lab_ = [(ΔL*)2 + (Δa*)2 + (Δb*)^2^]^1/2^ (1)

where L represents lightness, a the red-green axis, and b the yellow-blue axis.

ΔE_00_ was calculated using the CIEDE2000 formula, which refines perceptual uniformity over the traditional CIELAB system. The equation is as follows:

$${\Delta E}_{00}=\sqrt{\left[{\left(\frac{\Delta L^{\prime }}{K_{L}S_{L}}\right)}^{2}+{\left(\frac{\Delta C^{\prime }}{K_{C}S_{C}}\right)}^{2}+{\left(\frac{\Delta H^{\prime }}{K_{H}S_{H}}\right)}^{2}+RT\left(\frac{\Delta C^{\prime }}{K_{C}S_{C}}\right)\left(\frac{\Delta H^{\prime }}{K_{H}S_{H}}\right)\right]\;}$$

In this formula, *ΔL*′, *ΔC*′, and *ΔH*′ represent the differences in lightness, chroma, and hue between two samples. The rotation term (*R_T_*) introduces a correction factor that accounts for the interaction between chroma and hue differences, particularly influencing measurements in the blue region of the color space. Weighting functions (*S_L_*, *S_C_*, and *S_H_*) are applied to adjust the relative importance of lightness, chroma, and hue variations, reflecting the non-uniform perceptual sensitivity across the CIELAB color space. Additionally, parametric factors (*K_L_*, *K_C_*, and *K_H_*) are incorporated to compensate for differences in experimental conditions and observation parameters [21].

### Statistical Analysis

Statistical analyses were performed using SPSS software (version 23.0; IBM Corp., Chicago, IL, USA). The distribution of the data was assessed for normality using the Shapiro–Wilk test. For variables exhibiting normal distribution, comparisons between groups were conducted using the independent samples *t*-test; in cases where normality was not met, the Mann–Whitney U test was applied. Intragroup comparisons between baseline and post-immersion values were performed using paired samples *t*-tests for normally distributed data and Wilcoxon signed-rank tests for non-parametric data. A *p*-value of less than 0.05 was considered indicative of statistical significance.

## 3. Results

**Figure 3:**
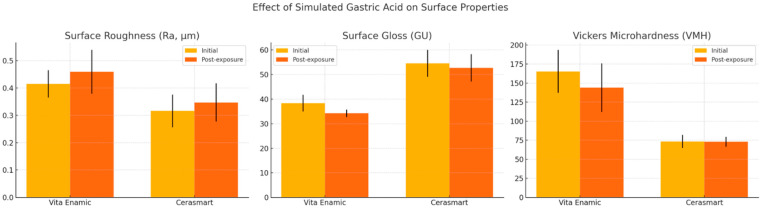
Comparison of surface roughness, gloss, and microhardness of Vita Enamic and Cerasmart before and after simulated gastric. Error bars represent standard deviations.

A detailed presentation of the experimental outcomes is provided in Table 2 and Figure 3.

### 3.1. Surface Roughness

**Figure 4:**
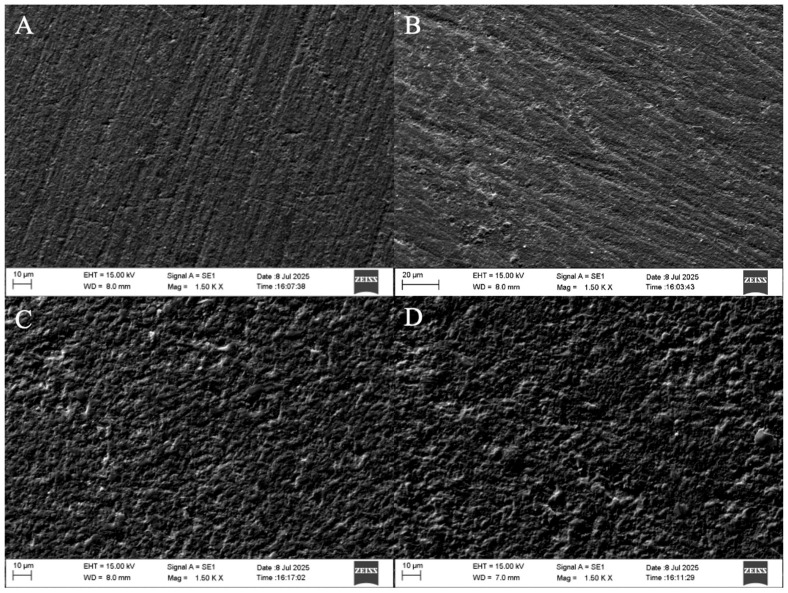
Representative SEM images (×1500 magnification) of the specimen surfaces before and after simulated gastric acid immersion. (**A**) Cerasmart before immersion; (**B**) Cerasmart after immersion; (**C**) Vita Enamic before immersion; (**D**) Vita Enamic after immersion.

A statistically significant increase in surface roughness was observed in both materials after immersion in simulated gastric acid (*p* = 0.025 for Vita Enamic; *p* = 0.044 for Cerasmart). Intergroup comparisons revealed that Cerasmart exhibited significantly lower surface roughness values compared to Vita Enamic at both baseline and post-exposure stages (*p* < 0.001 for both). It should be noted that both materials had Ra values above the clinically critical threshold of 0.2 μm, indicating a potential risk for increased plaque accumulation and secondary caries. These surface alterations were also confirmed by SEM images (Figure 4).

### 3.2. Surface Gloss

A significant reduction in surface gloss was observed for Vita Enamic after gastric acid exposure, decreasing from 38.3 ± 3.4 GU to 34.2 ± 1.5 GU (*p* < 0.001). In contrast, the reduction in gloss for Cerasmart (from 54.5 ± 5.5 GU to 52.7 ± 5.6 GU) did not reach statistical significance (*p* = 0.054). Intergroup comparisons revealed that Cerasmart consistently exhibited significantly higher gloss values than Vita Enamic at both time points (*p* < 0.001).

### 3.3. Vickers Microhardness

The microhardness of Vita Enamic significantly decreased after exposure to gastric acid (*p* = 0.001), whereas no significant change was detected in Cerasmart (*p* = 0.818). Notably, Vita Enamic presented significantly higher microhardness values than Cerasmart in both pre- and post-exposure measurements (*p* < 0.001 for both).

### 3.4. Color Stability

The mean color change values (ΔE_Lab_ and ΔE_00_) for both materials remained below the clinically perceptible threshold, with no statistically significant difference between Vita Enamic (ΔE_Lab_ = 1.18 ± 0.7, ΔE_00_ = 0.88 ± 0.84) and Cerasmart (ΔE_Lab_ = 1.13 ± 0.8, ΔE_00_ = 0.80 ± 0.79) (*p* = 0.491 and *p* = 0.290, respectively).

## 4. Discussion

This study aimed to evaluate the effects of simulated gastric acid exposure on the surface roughness, gloss, microhardness, and color stability of two hybrid CAD/CAM restorative materials—Vita Enamic (polymer-infiltrated ceramic network; PICN) and Cerasmart (resin nanoceramic). These materials were intentionally selected as representative examples of two fundamentally distinct hybridization strategies and are among the most widely used in contemporary clinical practice. Their inclusion allowed a comparative evaluation of how differences in polymer–ceramic integration influence material degradation under intrinsic acid challenges. Unlike earlier studies that typically focused on one or two isolated properties, the present investigation comprehensively assessed multiple clinically relevant parameters simultaneously. Moreover, by applying a 24 h continuous immersion protocol in hydrochloric acid at pH 1.2, this study modeled a worst-case scenario corresponding to prolonged and severe gastric reflux, thereby offering novel insights into material performance under extreme intraoral conditions.

The findings revealed that both materials exhibited discernible alterations in surface characteristics following gastric acid immersion; however, the magnitude and nature of these changes differed between the materials. Hence, the null hypothesis, which proposed that no differences would exist among the Hybrid CAD/CAM restorative material groups in the gastric acid medium, was rejected.

In the present study, specimen thickness was standardized at 2 mm to ensure both clinical relevance and adherence to established guidelines. According to ISO 4049 [22], polymer-based restorative materials should be prepared at a thickness of 2 mm, with a minimum threshold of 1.5 mm, to closely replicate the typical restorative layer dimensions encountered in clinical settings [23,24]. Consistent with this recommendation, numerous prior studies investigating the mechanical and surface characteristics of CAD/CAM restorative materials have employed specimen thicknesses of approximately 2 mm to facilitate methodological standardization and enable inter-study comparability [19,22]. Such a dimensional parameter provides an optimal compromise between simulating intraoral clinical conditions and maintaining uniform testing protocols.

Hydrochloric acid (HCl) with a pH of 1.2 was employed in this study to closely replicate the physicochemical properties of human gastric juice under physiological conditions. The selected pH value is within the established range of gastric acidity (pH 1.0–2.0), which is primarily maintained by hydrochloric acid secreted by the gastric parietal cells. The use of a pH of 1.2 aligns with established protocols in the dental materials literature aiming to simulate the intraoral exposure conditions associated with GERD and other acidogenic disorders [15,19]. This highly acidic environment is known to exert significant erosive effects on dental hard tissues and restorative materials, thereby serving as a valid model for assessing their chemical durability. Moreover, the application of pure HCl offers experimental standardization and reproducibility, mitigating the compositional variability encountered in alternative simulants such as artificial gastric juice or pepsin-containing solutions. Therefore, the use of HCl at pH 1.2 provides a clinically relevant and methodologically robust framework for evaluating the acid resistance of CAD/CAM restorative materials under simulated extreme oral conditions.

There is currently no standardized protocol regarding the optimal duration or methodology for in vitro simulation of gastric acid exposure to accurately reflect clinical conditions. Literature reports show variability in the estimated equivalency between immersion time and real-life exposure [16]. In their 2005 study, Abbate-Daga et al. [25] reported an average vomiting frequency of seven episodes per week in patients with moderate to severe bulimia. Assuming a minimum gastric juice contact time of 30 s per episode, this corresponds to approximately 3 h of cumulative exposure per year. Based on this calculation, 6 h of immersion in the present study simulates approximately 2 years of clinical exposure, while 24 h may represent about 8 years, as suggested in some studies [19,26]. Conversely, other investigations have proposed that a 45 min exposure could mimic the effects of approximately one month of clinical exposure to gastric acid [27]. Cengiz et al. [28] further suggested that a continuous 24 h immersion period should be regarded as a worst-case clinical scenario, particularly for individuals with severe GERD. In line with this rationale, the present study employed a 24 h immersion protocol to represent an extreme, long-term exposure condition.

The increase in surface roughness observed in both materials after immersion aligns with prior investigations reporting erosive effects of acidic environments on resin-based and ceramic materials. Cengiz et al. [28] similarly reported a significant increase in surface roughness of laboratory composites following exposure to simulated gastric juice, attributing this to the hydrolytic degradation of the polymer matrix and filler-matrix interface. Hybrid CAD/CAM restorative materials, particularly those incorporating a polymer network, are susceptible to water absorption and subsequent plasticization or hydrolysis when exposed to low pH conditions [19]. The significantly lower roughness values of Cerasmart compared to Vita Enamic at both baseline and post-immersion stages may be attributed to its nano-filled structure and more homogeneous distribution of fillers, which could provide enhanced resistance to acid-induced degradation. In parallel, studies conducted by Eğilmez et al. [29] and Cruz et al. [11] utilizing scanning electron microscopy (SEM) have demonstrated the presence of micropores and microcracks in Vita Enamic following exposure to gastric acid, further substantiating its pronounced susceptibility to acidic degradation.

According to the literature, maintaining surface roughness (Ra) values below 0.2 μm is considered critical for minimizing bacterial adhesion and subsequent biofilm formation, which are key contributors to secondary caries development [30]. Surface topography exceeding this threshold has been shown to facilitate microbial colonization by providing increased surface area and micro-retentive sites for plaque accumulation. In the present study, both hybrid CAD/CAM restorative materials—Vita Enamic and Cerasmart—demonstrated baseline and post-immersion Ra values that exceeded this clinically accepted limit, indicating a potential predisposition to biofilm formation. Interestingly, Pîrvulescu et al. reported even higher roughness values for both materials following hydrochloric acid exposure, with Ra increasing from 0.92 to 0.98 μm for Vita Enamic and from 0.89 to 0.92 μm for Cerasmart—nearly twice the values observed in the current study. This discrepancy underscores the critical role of polishing protocols and material-specific characteristics in achieving clinically acceptable surface smoothness [12].

These findings suggest that despite their advanced material compositions, these hybrid ceramics may not achieve an optimally smooth surface when subjected solely to intraoral polishing protocols. The elevated roughness values may reflect inherent microstructural characteristics of the materials, such as heterogeneous filler distribution or matrix composition, which may limit their polishability using conventional chairside techniques. These results underscore the potential need for alternative or supplementary finishing procedures—such as laboratory-based polishing or glazing—to ensure that the final surface texture meets clinical thresholds for long-term biological compatibility and esthetic performance.

An increase in the surface roughness of restorative materials not only compromises their mechanical durability by promoting uneven stress distribution but also has significant clinical implications regarding biofilm formation [13]. Quirynen et al. [31] demonstrated a clear correlation between increased surface roughness and heightened plaque accumulation, thereby linking surface topography to secondary caries risk and periodontal health. Moreover, surface irregularities can alter the optical behavior of a restoration by scattering light in a non-uniform manner, which adversely impacts surface gloss and perceived color. Surface gloss, a key esthetic parameter, is strongly influenced by the microtexture of the material. According to the American Dental Association (ADA) Professional Product Review, a gloss range of 40–60 GU is generally considered clinically acceptable for dental restorations [16]. Nevertheless, perceptibility and acceptability thresholds for surface gloss remain variable, depending on subjective evaluations, measurement instruments, and coordinate systems. A previous study reported these thresholds to range between 6.4 and 35.7 GU, underscoring the difficulty in establishing a universal standard for clinically significant gloss changes [32]. In the present study, although the reduction in gloss remained below the perceptibility threshold, only Cerasmart maintained gloss values within the ADA-recommended range, suggesting superior esthetic stability under acidic conditions.

Vita Enamic showed a statistically significant reduction in gloss after acid exposure, consistent with the increased surface roughness. Surface gloss and roughness are inversely correlated, as irregularities on the material surface lead to diffuse rather than specular light reflection [29]. In contrast, although Cerasmart also exhibited a decrease in gloss, the change was not statistically significant. This suggests that the smoother initial surface and potentially more stable resin matrix of Cerasmart confer better resistance to gloss deterioration under acidic conditions. The differences in behavior between Vita Enamic and Cerasmart can be further explained by considering confounding factors such as polymer type, filler composition, and water sorption tendencies. These structural and compositional differences directly influence water sorption behavior and chemical durability. Venz and Dickens evaluated the hydrophilicity of various dental monomers and reported a descending order of water sorption: TEGDMA > Bis-GMA > UDMA > HDMDMA [33]. This was attributed to the higher concentration of polar functional groups, such as hydroxyl, ether, and ester linkages, in more hydrophilic monomers. Accordingly, the relatively stable gloss values observed in Cerasmart may be due in part to its incorporation of urethane dimethacrylate (UDMA), a less hydrophilic monomer, which confers greater resistance to water sorption and subsequent surface degradation.

Color stability is a critical factor influencing the long-term esthetic performance of restorative materials and is closely interrelated with surface characteristics such as roughness and gloss. Increased surface roughness can lead to enhanced water sorption and superficial degradation [12] both of which may compromise the material’s color stability. Likewise, diminished surface gloss may reflect underlying surface irregularities that contribute to staining susceptibility. In the present study, both hybrid CAD/CAM restorative materials demonstrated ΔE values below the clinically perceptible threshold of 3.3, with no statistically significant differences between the groups. These findings indicate that, despite the observed alterations in surface topography and gloss, both materials maintained clinically acceptable resistance to discoloration following exposure to simulated gastric acid. This outcome is consistent with the findings of Pîrvulescu et al., who reported comparable ΔE values for both materials (ΔE = 0.71 for Cerasmart and ΔE = 0.81 for Vita Enamic) following 18 h of immersion in hydrochloric acid [12]. Similarly, Cengiz et al. documented minimal yet measurable color changes in composite materials exposed to acidic environments, attributing these alterations primarily to superficial matrix degradation and increased water sorption rather than intrinsic pigment penetration [28].

Microhardness testing revealed a significant decline in the Vickers microhardness values of Vita Enamic after exposure, whereas Cerasmart remained unaffected. This finding is particularly noteworthy, as hardness is a key indicator of a material’s resistance to wear and surface deformation. The higher baseline hardness of Vita Enamic, attributable to its dominant ceramic network, may paradoxically render it more brittle and prone to surface softening under acidic conditions. Previous studies have shown that acid-induced demineralization and matrix degradation contribute to microhardness loss in both ceramics and composites [10,18,29,34]. In this context, the present findings align closely with those of Gulakar et al., who reported that the microhardness of Vita Enamic decreased from 165.38 to 133.13 after 96 h of exposure to hydrochloric acid at pH 1.2 [20]. In this study, the initial microhardness of Vita Enamic (165.4) declined to 144.1 following 24 h of exposure under similar pH conditions. As anticipated, the shorter exposure duration resulted in a comparatively smaller reduction in microhardness, thereby supporting the hypothesis that acid-induced softening of hybrid ceramics is both material-dependent and time-sensitive. These findings are further corroborated by the study of Pîrvulescu et al., who similarly observed a statistically significant decrease in the microhardness of Vita Enamic following 18 h of hydrochloric acid exposure, while Cerasmart exhibited no significant change [12].

The observed reduction in microhardness in Vita Enamic holds substantial clinical relevance, as it indicates increased susceptibility to mechanical wear, surface loss, and potential restoration failure under functional occlusal forces. Such degradation may compromise occlusal morphology, marginal integrity, and esthetic outcomes over time, particularly in patients presenting with conditions associated with chronic acid exposure, such as GERD or eating disorders. In contrast, the relative stability of Cerasmart under identical experimental conditions may be attributed to its higher polymer content, which allows for limited swelling without substantial structural breakdown. Cerasmart’s stable microhardness values thus suggest a potentially longer clinical service life, preserving both functional integrity and esthetic performance in environments characterized by persistent acidic challenges.

The greater susceptibility of Vita Enamic to surface degradation following simulated gastric acid exposure can be attributed to its unique dual-network structure, consisting of a dominant feldspathic ceramic matrix (86% by weight) infiltrated with a low-viscosity polymer phase composed primarily of UDMA and triethylene glycol dimethacrylate (TEGDMA) [12,20]. This microstructural composition, while designed to combine the favorable mechanical properties of ceramics and polymers, presents inherent vulnerabilities when exposed to highly acidic environments. The feldspathic ceramic phase is particularly susceptible to acid-induced dissolution, which compromises the integrity of the surrounding polymer-infiltrated network. As the ceramic matrix dissolves, the interface between the ceramic and polymer components becomes more pronounced, potentially resulting in surface heterogeneity and increased roughness. Furthermore, SEM observations in this (Figure 4) as well as previous studies have identified the formation of microcracks and microporosities on the surface of Vita Enamic post-immersion, suggesting that the acidic medium not only erodes the material superficially but also propagates structural defects [19,20]. These findings collectively support the notion that the ceramic-dominant network of Vita Enamic, while beneficial for hardness, renders it more vulnerable to surface deterioration under low pH conditions compared to polymer-rich materials such as Cerasmart.

Collectively, these findings have important clinical implications. In patients with chronic acid exposure—such as those with GERD or eating disorders—Cerasmart may be the preferred restorative material due to its superior resistance to surface degradation, gloss reduction, and microhardness loss. However, these results cannot be generalized to all hybrid or conventional CAD/CAM materials, given the influence of compositional and microstructural variability on clinical performance. Clinicians should also consider adjunctive strategies, including protective coatings, topical fluoride, neutralizing rinses, and patient education to mitigate the impact of gastric acid on restorations and preserve long-term esthetic and functional outcomes.

This study has several limitations that should be acknowledged. First, its in vitro design does not fully replicate the complex and dynamic oral environment, where fluctuations in pH, salivary buffering capacity, enzymatic activity, mechanical abrasion from mastication and toothbrushing, and intraoral temperature variations play significant roles in material degradation. In particular, the absence of thermomechanical cycling and brushing simulation restricts the ability to reproduce the functional and mechanical stresses that restorations are routinely subjected to in vivo. Moreover, reliance on a single immersion protocol with fixed exposure durations may not adequately capture the variability in gastroesophageal reflux patterns, vomiting frequency, or dietary acid challenges encountered in clinical practice.

Another limitation is that only CAD/CAM restorative materials —Vita Enamic and Cerasmart— were evaluated. Although these materials are among the most widely utilized and clinically relevant representatives of polymer-infiltrated ceramic-based and resin nanoceramic materials, respectively, other CAD/CAM materials with distinct microstructural compositions and manufacturing processes exist. The exclusion of these alternatives restricts the generalizability of the present findings, as each product may exhibit distinct responses to acidic challenge. Future investigations should therefore incorporate a broader spectrum of CAD/CAM materials—including additional polymer-infiltrated ceramics, resin nanoceramics, and conventional ceramics—while employing experimental designs that more accurately approximate clinical conditions, such as dynamic pH fluctuations, temperature variations, extended immersion protocols, and thermomechanical loading. Studies assessing the effects of protective surface coatings and preventive agents would further enhance the clinical relevance and external validity of the findings.

Based on the findings of this study, it can be recommended that clinicians prioritize the selection of restorative materials with high chemical and mechanical stability in acidic environments, such as hybrid ceramics characterized by low initial surface roughness and enhanced resistance to erosive challenges. Additionally, the application of protective coatings or sealants on restoration surfaces may effectively reduce acid-induced damage; however, these interventions require periodic evaluation and renewal to maintain their protective function. Patient education regarding the detrimental effects of gastric acid and the encouragement of dietary and behavioral modifications to minimize acid exposure are essential preventive measures. Furthermore, the use of neutralizing rinses immediately after acid contact and topical fluoride treatments may further improve the resistance of both restorative materials and the underlying dental tissues.

## 5. Conclusions

Within the limitations of this in vitro study, the following conclusions can be drawn regarding the effects of simulated gastric acid exposure on hybrid CAD/CAM restorativematerials:

- Simulated gastric acid exposure adversely affected surface properties, particularly increasing surface roughness and reducing gloss in both materials.
- Vita Enamic showed more pronounced degradation, likely due to its higher feldspathic ceramic content, and the increased susceptibility of its ceramic–polymer interface to acidic conditions.
- Cerasmart exhibited greater surface stability, which may be attributed to its homogeneous nano-filled structure, and the presence of a UDMA-based resin matrix.
- Despite surface changes, both materials maintained color changes within clinically acceptable limits, suggesting adequate resistance to discoloration under acidic conditions.

These results highlight the critical role of material composition in determining the long-term esthetic durability of CAD/CAM restorations exposed to acidic environments. Selection of polymer-rich, acid-resistant materials such as Cerasmart may confer a clinical advantage in maintaining both surface integrity and esthetic outcomes under erosive conditions.

## Figures and Tables

**Table 1:** Mechanical properties and filler components of the materials used in the study.

Classification	Material	Composition	Flexural Strength	Elasticity Modulus	Manufacturer	LOT
Polymer-infiltrated ceramic, Hybrid CAD/CAM block	Vita Enamic	86% feldspathic ceramic (primarily SiO_2_, Al_2_O_3_, Na_2_O, K_2_O, and other oxides), 14% polymer (UDMA, TEGDMA)	150–160 Mpa	30 Gpa	Vita Zahnfabrik, Essen, Germany	205260
Resin Nanoceramics, Hybrid CAD/CAM block	Cerasmart	UDMA, Bis-MEPP, DMA, 71% silica, barium glass	246 Mpa	9.6 Gpa	GC Corporation, Tokyo, Japan	2001286

Bis-MPEPP: Bisphenol A polyethoxy methacrylate, DMA: dimethacrylate, TEGDMA: triethyleneglycol dimethacrylate, UDMA: urethane dimethacrylate.

**Table 2:** Effects of simulated gastric acid on surface roughness, gloss, microhardness, and color of Vita Enamic and Cerasmart.

Materials	Surface Roughnes (Ra in µm)		*p*
	**Initial**	**Post-Exposure**	
Vita Enamic	0.415 ± 0.05	0.459 ± 0.08	**0.025** ′^,^*
Cerasmart	0.316 ± 0.06	0.347 ± 0.07	**0.044** ′^,^*
***p***	**<0.001** ^†^^,^*	**<0.001 ^‡^** **^,^** *	
	**Surface Gloss (In GU)**		
Vita Enamic	38.3 ± 3.4	34.2 ± 1.5	**<0.001** ″^,^*
Cerasmart	54.5 ± 5.5	52.7 ± 5.6	0.054 ′
***p***	**<0.001 ^‡^** **^,^** *	**<0.001 ^†^** **^,^** *	
	**Vickers Micro Hardness (VMH)**		
Vita Enamic	165.4 ± 28.2	144.1 ± 31.8	**0.001** ″^,^*
Cerasmart	73.5 ± 8.5	73.1 ± 6.6	0.818 ″
***p***	**<0.001 ^‡^** **^,^** *	**<0.001 ^‡^** **^,^** *	
	**Color Change (ΔE_Lab_)**	**Color Change (ΔE_00_)**	
Vita Enamic	1.18 ± 0.7	0.88 ± 0.84	
Cerasmart	1.13 ± 0.8	0.80 ± 0.79	
***p***	0.491 ^†^	0.290 ^†^	

‡: Independent Samples *t*-test; †: Mann–Whitney U test; ′: Wilcoxon *t*-test; ″: Paires samples *t*-test; * *p* < 0.05.

## Data Availability

The raw data supporting the conclusions of this article will be made available by the authors on request.

## Abbreviations

The following abbreviations are used in this manuscript:

ADA	American Dental Association
Bis-MEPP	Bisphenol A polyethoxy methacrylate
CAD/CAM	Computer-aided design and computer-aided manufacturing
DMA	Dimethacrylate
GERD	Gastroesophageal reflux disease
PICNs	Polymer-infiltrated ceramic networks
TEGDMA	triethyleneglycol dimethacrylate
UDMA	Urethane dimethacrylate
